# Maniform Presentation of Behavioral Variant Frontotemporal Dementia Mimicking Late-Onset Mania: A Diagnostic Challenge

**DOI:** 10.7759/cureus.118016

**Published:** 2026-10-16

**Authors:** Ana Aires, Mariana Lázaro

**Affiliations:** 1 Psychiatry, Unidade Local de Saúde do Algarve, Faro, PRT

**Keywords:** behavioral variant frontotemporal dementia, frontotemporal dementia, late-onset psychiatric symptoms, mania, neurodegeneration

## Abstract

Frontotemporal dementia (FTD) is a neurodegenerative disorder that may initially present with behavioral and affective symptoms, often mimicking primary psychiatric conditions, such as bipolar disorder, leading to frequent misdiagnosis.

We report the case of a 70-year-old woman with no prior psychiatric history who presented with a first episode of maniform behavior, characterized by euphoria, disinhibition, decreased need for sleep, impulsive spending, and socially inappropriate conduct, mimicking late-onset bipolar disorder. Neuropsychological evaluation revealed significant executive dysfunction. Brain magnetic resonance imaging demonstrated bilateral frontal and anterior temporal lobe atrophy consistent with behavioral variant FTD (bvFTD).

This case highlights the diagnostic challenge of distinguishing late-onset manic syndromes from neurodegenerative disease. It underscores the importance of integrating clinical history, cognitive assessment, and neuroimaging to improve diagnostic accuracy and avoid misdiagnosis.

## Introduction

Frontotemporal dementia (FTD) is a progressive neurodegenerative disorder characterized by predominant degeneration of the frontal and temporal lobes, resulting in behavioral, executive, and language impairments. The behavioral variant FTD (bvFTD) is the most common clinical phenotype and is typically associated with disinhibition, apathy, impulsivity, personality changes, and executive dysfunction [1,2].

Early manifestations of bvFTD may closely resemble primary psychiatric disorders, particularly bipolar disorder and manic syndromes, often leading to misdiagnosis and delayed diagnosis. Previous studies have reported that a substantial proportion of patients with bvFTD are initially diagnosed with a psychiatric disorder [3,4]. This diagnostic challenge is particularly relevant in older adults presenting with a first episode of manic-like symptoms, including euphoria, disinhibition, decreased need for sleep, impulsive spending, and socially inappropriate behavior. In such patients, a progressive course, persistent behavioral changes, executive dysfunction, impaired insight, and functional decline should raise suspicion of an underlying neurodegenerative disorder. These features may help distinguish bvFTD from late-onset bipolar disorder, particularly when typical affective episodes are absent or do not fully explain the overall clinical picture.

The revised Rascovsky criteria provide a structured framework for the diagnosis of bvFTD, distinguishing between possible, probable, and definite bvFTD according to the presence and progression of characteristic behavioral and cognitive features, functional decline, and supportive neuroimaging findings [4]. The present case illustrates the diagnostic overlap between a late-onset maniform presentation and bvFTD and highlights the importance of longitudinal clinical assessment, neuropsychological evaluation, and neuroimaging in distinguishing neurodegenerative disease from primary psychiatric disorders.

## Case presentation

A 70-year-old woman with no prior psychiatric or neurological history presented with a two-month history of progressive behavioral changes characterized by elevated mood, disinhibition, reduced need for sleep, impulsive spending, and socially inappropriate behavior. According to family members, symptoms evolved progressively rather than episodically, with increasing impairment in judgment and loss of emotional regulation.

On mental status examination, she was euphoric, overly familiar, distractible, and exhibited pressured, tangential speech with severely impaired insight. No hallucinations or structured delusional content were present. Although the initial impression was consistent with late-onset mania, subtle cognitive deficits were evident during clinical interaction.

Executive function was further assessed using the Frontal Assessment Battery (FAB), validated by Dubois et al. [5], which revealed a score of 8/18, consistent with significant frontal dysfunction.

Neuropsychological testing demonstrated impaired cognitive flexibility, inhibitory control, abstract reasoning, and social cognition, particularly theory of mind deficits. Routine laboratory studies, including metabolic, infectious, and endocrine panels, were normal, reducing the likelihood of secondary systemic causes.

Brain magnetic resonance imaging revealed bilateral frontal lobe atrophy with orbitofrontal predominance and anterior temporal atrophy with relative posterior cortical sparing, consistent with a pattern suggestive of bvFTD (Figure 1) [6].

**Figure 1:**
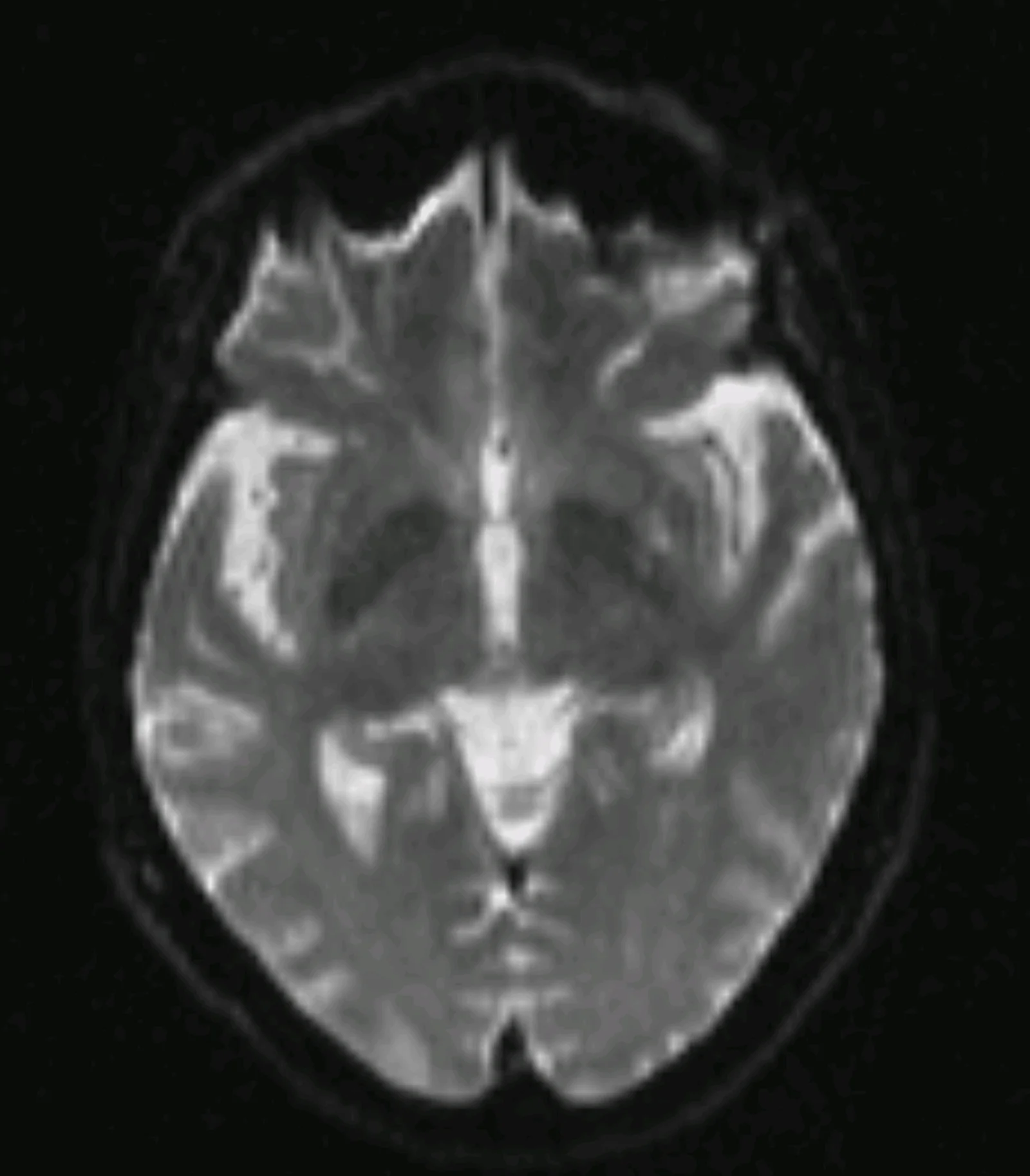
Axial brain MRI demonstrated bilateral frontal and anterior temporal lobe atrophy, predominantly involving the orbitofrontal regions. No focal vascular lesions, mass lesions, hydrocephalus, or significant hippocampal atrophy were identified.

The patient was initially started on low-dose antipsychotic treatment with quetiapine (up to 100 mg/day), with partial improvement in sleep and agitation. Given the progressive nature of symptoms and emerging neuroimaging findings, pharmacological management was subsequently reoriented toward symptomatic and supportive care, with gradual discontinuation of antipsychotic escalation due to minimal sustained response.

Non-pharmacological interventions were implemented, including psychoeducation of family members, behavioral management strategies, and caregiver support to address increasing functional dependence and social disinhibition. Despite these measures, the patient showed continued gradual decline in executive functioning and social cognition over time, consistent with a neurodegenerative trajectory [2,7].

At six-month follow-up, there was further deterioration in behavioral regulation, with increased apathy interspersed with disinhibited episodes, progressive loss of autonomy in instrumental activities of daily living, and worsening executive dysfunction. The overall clinical evolution, together with neuropsychological and neuroimaging findings, reinforced the diagnosis of bvFTD, and care was transitioned to a structured multidisciplinary follow-up focused on symptom management and caregiver support.

## Discussion

This case highlights the diagnostic complexity and frequent overlap between bvFTD and primary psychiatric disorders, particularly late-onset bipolar disorder. Early bvFTD may present with prominent behavioral dysregulation, including mood elevation, increased activity, disinhibition, impulsivity, and socially inappropriate behavior, closely mimicking manic or hypomanic episodes [2,3,7].

Late-onset bipolar disorder was initially considered because of the elevated mood and behavioral activation. However, the absence of episodic remission, progressive and continuous behavioral deterioration, and early executive dysfunction were inconsistent with a primary affective disorder. Unlike bipolar disorder, which typically follows an episodic course with periods of remission, bvFTD follows a progressive trajectory with persistent decline in cognitive, behavioral, and social functioning [3].

Other causes of late-onset behavioral syndromes were also considered. Alzheimer’s disease was less likely because there was no early prominent amnestic syndrome and frontal behavioral symptoms predominated. Dementia with Lewy bodies was considered less likely in the absence of characteristic features such as visual hallucinations, fluctuating cognition, or parkinsonism. Vascular cognitive impairment and structural brain disease were also considered, particularly given the patient's age, but the clinical and neuroimaging pattern was more consistent with bvFTD. Autoimmune or infectious encephalitis and metabolic or endocrine disorders were considered and investigated where clinically appropriate, with no alternative cause identified. Medication- or substance-induced behavioral symptoms and delirium were also considered; the absence of an appropriate temporal relationship or an acute fluctuating course made these diagnoses less likely. The convergence of the progressive clinical course, neuropsychological impairment, and neuroimaging findings supported a diagnosis of probable bvFTD [1,2,3,6].

Neuropsychological assessment demonstrated significant executive dysfunction, consistent with frontal lobe impairment. Deficits in cognitive flexibility, inhibitory control, and social cognition are characteristic of bvFTD and may precede more global cognitive decline [1,6,7]. Neuroimaging further supported the diagnosis, revealing bilateral frontal and anterior temporal atrophy, a pattern consistent with established diagnostic frameworks for bvFTD [6].

Neuroanatomically, bvFTD is associated with degeneration of the orbitofrontal cortex, ventromedial prefrontal cortex, and anterior temporal regions, contributing to impaired judgment, loss of empathy, and behavioral disinhibition. Dysfunction of large-scale networks, including the salience network, may help explain maniform presentations in the absence of primary mood pathology [7]. The revised Rascovsky diagnostic criteria emphasize the integration of progressive behavioral or cognitive deterioration, characteristic behavioral features, executive dysfunction, functional decline, and supportive neuroimaging findings. In the present case, these findings supported classification as probable bvFTD [3,4]. Nevertheless, early differentiation from psychiatric disorders remains challenging, particularly when behavioral symptoms predominate. Misdiagnosis may lead to inappropriate pharmacological treatment, delayed recognition of neurodegenerative disease, and increased caregiver burden [8].

Routine cognitive assessment and consideration of neuroimaging should therefore be encouraged in older adults presenting with a first manic or hypomanic episode, particularly in the absence of previous psychiatric history or when the clinical course is progressive or atypical. Emerging biomarkers, such as neurofilament light chain (NfL), may further assist diagnostic differentiation between psychiatric and neurodegenerative disorders, although their routine clinical application remains limited [7,8].

## Conclusions

bvFTD may present with prominent maniform symptoms that closely resemble late-onset bipolar disorder, particularly in older adults without prior psychiatric history. In this clinical context, the emergence of first-episode manic-like behavior should prompt consideration of an underlying neurodegenerative process. Progressive behavioral change, early and disproportionate executive dysfunction, and characteristic frontal and anterior temporal atrophy on neuroimaging are key features that help distinguish bvFTD from primary psychiatric disorders. As illustrated in this case, reliance solely on psychiatric symptomatology may lead to misdiagnosis and inappropriate treatment, particularly in the early stages of disease. This case underscores the importance of a multidisciplinary diagnostic approach integrating clinical assessment, neuropsychological testing, and neuroimaging findings. Such integration is essential for improving diagnostic accuracy and is consistent with established diagnostic frameworks, including the Rascovsky criteria for bvFTD.

Early recognition of bvFTD has important clinical implications, including avoidance of unnecessary exposure to psychotropic medications that are unlikely to modify the underlying neurodegenerative disease, timely implementation of supportive care, and appropriate caregiver counseling. Early diagnosis also facilitates advance care planning and enables patients and families to better prepare for progressive functional decline. Overall, this case reinforces the need for a high index of suspicion for neurodegenerative disease in atypical late-onset psychiatric presentations.

## References

[1] Warren JD, Rohrer JD, Rossor MN (2013). Clinical review. Frontotemporal dementia. BMJ.

[2] Olney NT, Spina S, Miller BL (2017). Frontotemporal dementia. Neurol Clin.

[3] Ducharme S, Dickerson BC (2015). The neuropsychiatric examination of the young-onset dementias. Psychiatr Clin North Am.

[4] Rascovsky K, Hodges JR, Knopman D (2011). Sensitivity of revised diagnostic criteria for the behavioural variant of frontotemporal dementia. Brain.

[5] Dubois B, Slachevsky A, Litvan I, Pillon B (2000). The FAB: a Frontal Assessment Battery at bedside. Neurology.

[6] Whitwell JL, Josephs KA (2012). Neuroimaging in frontotemporal lobar degeneration—predicting molecular pathology. Nat Rev Neurol.

[7] Seelaar H, Rohrer JD, Pijnenburg YA, Fox NC, van Swieten JC (2011). Clinical, genetic and pathological heterogeneity of frontotemporal dementia: a review. J Neurol Neurosurg Psychiatry.

[8] Woolley JD, Khan BK, Murthy NK, Miller BL, Rankin KP (2011). The diagnostic challenge of psychiatric symptoms in neurodegenerative disease: rates of and risk factors for prior psychiatric diagnosis in patients with early neurodegenerative disease. J Clin Psychiatry.

